# Wearable technology for mobility measurement in orthopedics and traumatology: a comparison of commercially available systems

**DOI:** 10.1007/s00402-025-05803-1

**Published:** 2025-03-15

**Authors:** A. M. Keppler, R. Zaccaria, M. Weigert, L. Keppler, W. Böcker, C. Neuerburg, R. Schniepp, J. Fürmetz

**Affiliations:** 1 Department for Orthopaedics and Trauma Surgery, Musculoskeletal University Center Munich (MUM), University Hospital, LMU Munich, Marchioninistrasse 15, Munich, 81377 Germany; 2 Department of Neurology, University Hospital, LMU Munich, Marchioninistrasse 15, Munich, 81377 Germany; 3 Department of Trauma Surgery, Trauma Center Murnau, Murnau, 82418 Germany

**Keywords:** Mobility, Orthogeriatric, Gait, Lower limb injuries

## Abstract

**Background:**

Wearable activity sensors offer valuable insights into physical activity and are increasingly used in clinical and rehabilitation settings. However, most are designed for healthy individuals, necessitating a thorough evaluation of their applicability for pathological gait patterns. This study aims to assess the accuracy of commercially available wearables in measuring gait patterns among patients with lower limb injuries compared to healthy individuals.

**Methods:**

A prospective cohort study enrolled 40 participants divided into four groups: Group A (younger patients with lower limb injuries with age < 75y), Group B (younger healthy individuals with age 75y), Group C (elderly patients with lower limb injuries and age 75y), and Group D (elderly healthy individuals with age > 75y). Mobility was assessed in real-world scenarios using four wearable devices (Apple Watch Series 4, Fitbit Charge 3, ActivPal 4, and StappOne Insoles V1.0) across three gait speeds in a gait laboratory, with GAITrite mats and video as gold standards.

**Results:**

Accuracy varied significantly between devices. The accelerometer-based wearables (Apple Watch Series 4, Fitbit Charge 3 and ActivPal 4™) underestimated cumulative step count compared to pressure-based Stappone v1. 0, especially for slow and restricted gait patterns (Groups C and D). Relative Difference of Wearables Measurements to the true numbers of steps (Group C: AW -21.83%, FB -28.99%, AP -20.00% versus SO 0.00% - Group D: AW -8.51%, FB -14.29%, AP -20.00% versus SO 4.55%). Zero measurements occurred frequently with wrist-worn devices, highlighting their limitations in detecting slow or restricted movements. In contrast, pressure-based StappOne Insoles demonstrated superior accuracy, with minimal deviations across all groups and gait speeds. The inaccuracy was exacerbated by factors such as the use of mobility aids, partial weight-bearing, and postoperative restrictions, which altered arm and leg movements.

**Conclusions:**

Accelerometer-based wearables require algorithmic improvements to address the challenges of slow and pathological gait patterns. The frequent occurrence of zero measurements with wrist-worn devices underscores their limited utility in clinical populations. Practical challenges, such as altered movement patterns due to mobility aids and partial weight-bearing, further limit their accuracy. Pressure-based systems, while accurate, face practicality issues for daily use. These findings emphasize the need for tailored wearable technologies for orthopedic and trauma patients.

**Level of evidence:**

Prospective cohort study, Level of Evidence 2.

## Introduction

Restoration of mobility is one of the overarching treatment goals in conservative and surgical therapy regimens for orthogeriatric patients. In-hospital mobilization and a rapid return to “physiologic” daily mobility after therapy improve direct and indirect health outcomes such as mortality, disability, pain, depression, and social isolation, which ultimately reduce quality of life [1–3]. Even in younger patients, a rapid return to activity and exercise capacity should be ensured, as rapid mobilization not only has positive individual health aspects, but also has health economic benefits, as Bouman et al. demonstrated in multitrauma patients [4].

In this context, the quantitative assessment of gait and mobility patterns appears to be crucial and offers the possibility of tailoring treatment to patient-oriented outcomes according to the World Health Organisation (WHO) concept of functionality, disability and health.

Today, developments in wearables or also called physical activity monitors (PAMs) offer promising opportunities for quantifying movement. Most devices are small, lightweight and wireless, allowing them to be worn without being intrusive. In addition, their long battery life allows them to collect, analyze and transmit data in a real-world environment.

Combined with automatic data processing techniques for continuous, multimodal motion data, fast and reliable segmentation and data analysis for gait and mobility patterns seems realistic [5]. Nearly every smartphone contains a motion sensor, and the growing number of smartwatches and related applications are increasing public awareness of these technologies [6, 7]. During the COVID-19 pandemic, this development accelerated significantly. Many approaches have been developed to study user mobility patterns using wearables [8]. With the increasing use of fast-track approaches and improved rehabilitation programs in arthroplasty, patients are being discharged earlier from inpatient care [9–11]. Several providers are already trying to improve follow-up care through PAMs, offering patients remote care or personalized rehabilitation through apps and smart devices [12]. Zimmer Biomet is currently conducting a multi-center study of Apple Watch monitored knee and hip replacement follow-up.(Clinical Trial Number: NCT03737149).

These developments make it even more important for the clinician to know the accuracy of the measurements. Most PAMs are based on accelerometry and have been validated in young, healthy patients without gait impairment.

On the one hand, accelerometers offer advantages such as robustness, compact design and long battery life, but on the other hand, the accuracy depends only on the algorithm used [13]. In addition to higher resolution of up to 100 Hz, newer systems have additional sensors such as gyroscopes, GPS, or pressure sensors. However, it is unclear to what extent multiple sensors improve accuracy in complex gait patterns. For slow gait speeds, such as those seen in the elderly and injured, accelerometer-based algorithms show inadequate step and gait speed detection [13–15]. Walking with crutches makes it difficult to register step counts with PAMs [16]. Here, pressure-sole-based wearables could be an interesting approach to better assess the detectives in patients with gait disorders caused by trauma. With the help of such pressure-based soles, a large amount of data can be collected during gait [17].Insole measurements provide more detailed gait parameters than accelerometry-based systems in elderly trauma patients, but are inconvenient for daily use. To our knowledge, commercially available PAMs have not been validated in lower extremity trauma patients of different ages. This gap in understanding wearable accuracy under pathological, postoperative conditions is critical. While research has explored wearables in healthy populations, limited evidence exists on their performance among patients with lower extremity injuries or age-related gait impairments. To address this, our study evaluates the accuracy of four commercially available wearables in diverse patient groups across multiple gait speeds, with a focus on identifying limitations and potential improvements in their application to clinical settings.

The PAMs used in this study differ in terms of the technical sensor equipment as well as in the way they are worn. The results of this study are intended to provide information on the potential errors in the use of the investigated PAMs for short- and long-term recording of physical activity, especially in orthopedic or trauma patients with limited gait parameters. For digitalized, app-based rehabilitation, a precise understanding of “what” is being measured using such devices is also essential for doctors and therapists.

## Methods

### Study design and participants

#### Prospective cohort study, level of evidence 2

This study was registered and approved by the local ethics committee (file number: 627 − 16). The study adhered to the tenets of the Declaration of Helsinki. The datasets generated and/or analyzed during the current study are not publicly available due to data protection regulations, but are available from the corresponding author upon reasonable request.

Inclusion criteria (see Table 1 for details) were consecutively enrolled between February 2020 and December 2020 at a level I trauma center with specialized orthogeriatric care. Patients were excluded from the study if the following conditions were present at the time of enrolment: immobility before surgery (bedridden patients, severe neurological disorders), dementia (Minimal Mental State Exam (MMSE) lower than 27), delirium, language barrier and polytrauma and/or external fixation. Informed consent was obtained from all individual participants included in the study. The patients completed gait analysis using the different sensor devices. The StappOne (Stappone, Sulz, Austria) insoles v1.0 sensors were fitted into the shoe of each participant according to their appropriate shoe size. The Apple Watch Series 4 (Operating System: watchOS 5) (Apple Inc., Cupertino, California, USA) was attached to the left and the Fitbit Charge 3 (Fitbit Charge 3 Inc., San Francisco, CA) to the right of each wrist. In addition, the ActivPal 4™ (PAL Health Technologies, IL, USA) micro PAL was applied to the right thigh, and the opposite side was used for patients who had a restriction on the right extremity due to the injury. All sensors were reset before each run. Surgical patients were measured between 4 and 7 days postoperatively. Patients who received conservative treatment for their fractures were assessed 4–7 days after diagnosis. A standardized pain medication regime was used for all patients according to WHO treatment guidelines. No peripheral regional pain catheter was used during gait analysis. All patients were allowed to use a walking aid of their choice during the measurement. The investigations were conducted in a gait laboratory. In addition to continuous video recording, the gait was simultaneously documented using GAITRite^®^ (Rölke Pharma GmbH, Hamburg, Germany) single-layer pressure-sensitive treadmill in a gait laboratory, with the track repeated twice for each speed.The gait analysis included a protocol with 3 different speed conditions: preferred, slow, and maximum speed. To evaluate the quality of the step detection, the subject’s gait was additionally videotaped with a software synchronized high-resolution camera (2D) and the steps were counted. Synchronization of all devices was achieved using a timestamp system. or the accelerometer-based devices, all data were cleared before each measurement to prevent errors, and the StappOne pressure insoles were calibrated for each participant according to the manufacturer’s specifications.

If there were differences between the video and GAITRite^®^ step counts, they were averaged. Patients were always protected by a researcher walking alongside.

**Table 1:** Study groups

	Age	Gait Impairment	*n*
Group A	18-75y	No Injury, no impairment	10
Group B	> 75y	No Injury, no Impairment	10
Group C	18-75y	Injury or fracture of the lower limb < 6 weeks	10
Group D	> 75y	Injury or fracture of the lower limb < 6 weeks	10

### Statistical analysis

The accuracy of step detection for the four investigated PAMs was evaluated using both descriptive and model-based approaches. Descriptive analysis focused on the percentage deviation between AT-measured and observed step counts, with the mean absolute percentage error (MAPE) calculated for each device. The model-based analysis employed a two-step strategy. First, a linear mixed-effects model with a random intercept for patients was used to assess step count accuracy for regular non-zero measurements. Covariates included device type, age group (young/old), condition (healthy/trauma), gait speed, and their interactions. Repeated measures under identical conditions were averaged as single observations. Second, zero measurements were analyzed using a mixed logistic regression model for the Apple Watch Series 4 and Fitbit Charge 3, incorporating a quadratic speed term to account for higher zero-measurement rates at extreme speeds. Likelihood-ratio tests determined the significance of effects (α = 0.05). Statistical analyses were performed using R (R-Foundation, Vienna, Austria), with models fitted via the lme4 package. Predicted values were visualized using sjPlot and ggeffects packages [18–20].

## Results

### Demographics

The mean age differed between the groups, but not to a significant degree. The demographic details of each group are shown in Table 2.

**Table 2:** Patient characteristics and demographic

Characteristic	Group A	Group B	Group C	Group D
BMI (kg/m²)	23,5 (± 2.2)	22,7 (± 3.3)	24,6 (± 3.0)	25,9 (± 4,7)
Age (years)	51,7 (± 12.8)	57.6 (± 16.4)	81.8 (± 3.2)	81 (± 4,7)
Body height (cm)	175,3 (± 9,8)	172,1 (± 10,7)	175,7 (± 8,2)	174,6 (± 5,5)
Female sex, n (%)	40	40	40	20

BMI: Body Mass Index; ANOVA-Test

### Missing values

Before considering the statistical analysis, it should be noted that in some groups it was not possible to complete the entire study protocol, especially at the maximum speed. Table 3 below shows the missing values for each group in total and as a percentage according to the walking speed.

**Table 3:** Missing values in the different groups at self-selected, slow and maximum speed

Patients/Speed	Selfchosen (%)	Slow (%)	Max (%)	Total (%)
Old/Trauma (%)	0.00	15.50	88.00	34.50
Old/Healthy (%)	5.50	5.00	95.00	35.17
Young/Trauma (%)	10.00	36.00	56.50	34.17
Young/Healthy (%)	0.00	0.50	12.50	4.33
Total (%)	3.88	14.25	63.00	27.04

In the last column, the total sum of missing values.It should be noted that especially the maximum speed was not completely done by each subject All values are shown in percent (%)

As can be seen in the table, measurements at maximum speed were largely omitted in the old-trauma (88.00%) and old-healthy (95.00%) groups due to the increased risk of falling early after surgery and the lack of physical resilience. Similarly, in the young patient group, almost half (56.50%) of the cases did not have the physical capacity to complete four runs at maximum speed early postoperatively.

Therefore, it should be noted that 27.04% of the measurements are missing in all the following graphs of the data set. This must be taken into account for the following statistical analysis and the significance of the results obtained. and will be discussed in more detail under “Limitations of the Study” in the Discussion section that follows.

### Zero measurements” for wrist-worn wearables

It is important to note that the results varied greatly depending on the location on the body where the devices were worn, as well as the speed of the walk. For example, the wrist-worn wearables, i.e., the Apple Watch Series 4 and Fitbit Charge 3, often did not detect any activity at all at slow speeds. The small arm swing of the “slow” subjects or those with walkers during these runs seemed to be insufficient to reach the threshold for detecting a step. This resulted in frequent zero measurements with unacceptable sensitivity and specificity for these devices. As shown in the table below, zero measurements occurred exclusively with the wrist-worn wearables, and at a relatively high rate. For example, the Apple Watch failed to record a total of 81 runs, or about 16.88% of the total data set. For the Fitbit Charge 3, there were as many as 129 runs, or 26.88% of the total data set (Table 4).

In the context of the statistical analysis, we therefore evaluated the collected data set both with and without “zero values”. For this reason, we extended the plot sections by a “Without Zero Measurements” section, which shows the same plots without “zero values”.

**Table 4:** Total number of zero measurements of the wearables

Wearable/zero measurements	Number	Percentage (%)
Apple	81.00	16.88
Fitbit	129.00	26.88
PAL	0.00	0.00
StappOne	0.00	0.00

The small arm swing of the “slow” subjects or those with walkers during these runs seemed to be insufficient to reach the threshold for detecting a step with wrist worn devices.: Numerical and percentage. Zero measurements occurred exclusively with Apple and Fitbit Charge 3

### Comparison of wearables

**Fig. 1:**
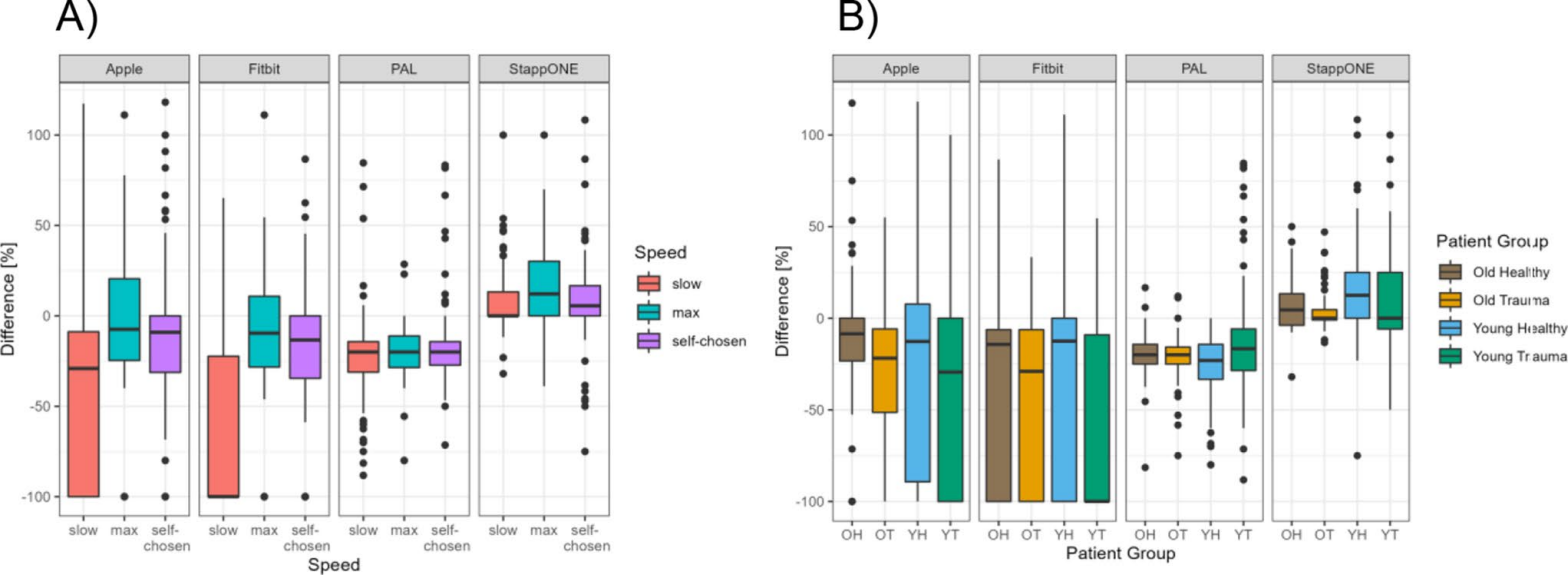
(**A**) Accuracy of the four different PAMs at different patients’ speeds, all results included. (**B**) The box plots describe the relative difference of steps recorded by the wearables to the actual number of steps depending on the patient group. Speed (Gl: slow gait speed; Gm: maximum gait speed, Gs: self-chosen gait speed). Patient Group (AG: Elderly (> 75 y.), healthy participants with free gait and no mobility impairment; AT Elderly (> 75 y.) patients with a lower limb injury or fracture; JG: Young (< 75 y.), healthy participants with free gait and no mobility impairment; JT Young (< 75 y.) participants with a fracture or injury to the lower limb that potentially alters the gait pattern)

Overall, the Apple Watch underestimated actual steps in the majority of runs. For example, the relative difference between the steps measured by the Apple Watch and reality was − 15.79%, with an interquartile range of -60.56–0.00%. The largest difference was seen at slow speed (-29.11%), with an interquartile range of -100% to -8.79% (Fig. 1). Significantly more accurate values were shown at self-selected speed (-9.09%) and especially at maximum speed (-7.42%). Similar to Fitbit, the Apple Watch also resulted in higher accuracy in healthy subjects (AG -8.51%; JG -12.70%; Fig. 1).

Similar to the Apple Watch, the Fitbit Charge 3 also underestimated the actual number of steps. The relative difference from reality was − 16.67%, with an interquartile range of -100.00–5.72% (Fig. 1). The largest deviation was also seen at slow speed, with a mean difference of -100.00% and an interquartile range of -100% to -22.35% (Fig. 1A). In particular, subjects with lower extremity injuries had a high mean inaccuracy (mean young trauma − 100.00%; mean old trauma − 28.99%) (Fig. 1B). Overall, there was an increase in measurement accuracy with increasing speed. For example, the mean difference was − 13.33% at self-selected speed and − 9.55% at maximum speed (Fig. 1A). This resulted in higher measurement accuracy in the healthy subjects (AG -14.29%; JG -12.50%).

The ActivPal also underestimated the actual number of steps (relative difference of 20.00%), but it had the lowest variance (UQ -27.27%; OQ -14.29%). In contrast to the other wearables, the measurement accuracy did not depend on the speed, the mean difference was the same at slow speed as well as at self-selected and maximum speed (Fig. 1A). (Fig. 1A). No relevant difference was found within the different groups (AG -20%, AT -20%, JG -23.08%, JT-16.67%) (Fig. 1B).

The StappOne insole showed the best agreement with the actual number of steps. Although these showed a discrete overestimation of steps on average at all gait speeds, they provided the highest measurement accuracy with respect to the actual number of steps in comparison. The relative difference was 3.85% with an interquartile range of 0.00–16.91% (Fig. 1A).

In contrast to the wrist-worn devices, the highest measurement accuracy was found at low speeds (median 0.00%) and decreased with increasing speed (self-selected speed 5.56% and maximum speed 12.14%).(Fig. 1B).

The wrist-worn wearables recorded so-called zero readings. To illustrate the individual error and accuracy without zero readings for the Apple Watch and Fitbit, the MAPE (mean absolute percentage error) was calculated using the following equation:

$$MAPE = {1 \over n}\sum\nolimits_{i = 1}^n {\left| {{{{m_i} - {t_i}} \over {{t_i}}}} \right|} $$

(m i = measured step count, t i = true step count and n= number of measurements)

**Fig. 2:**
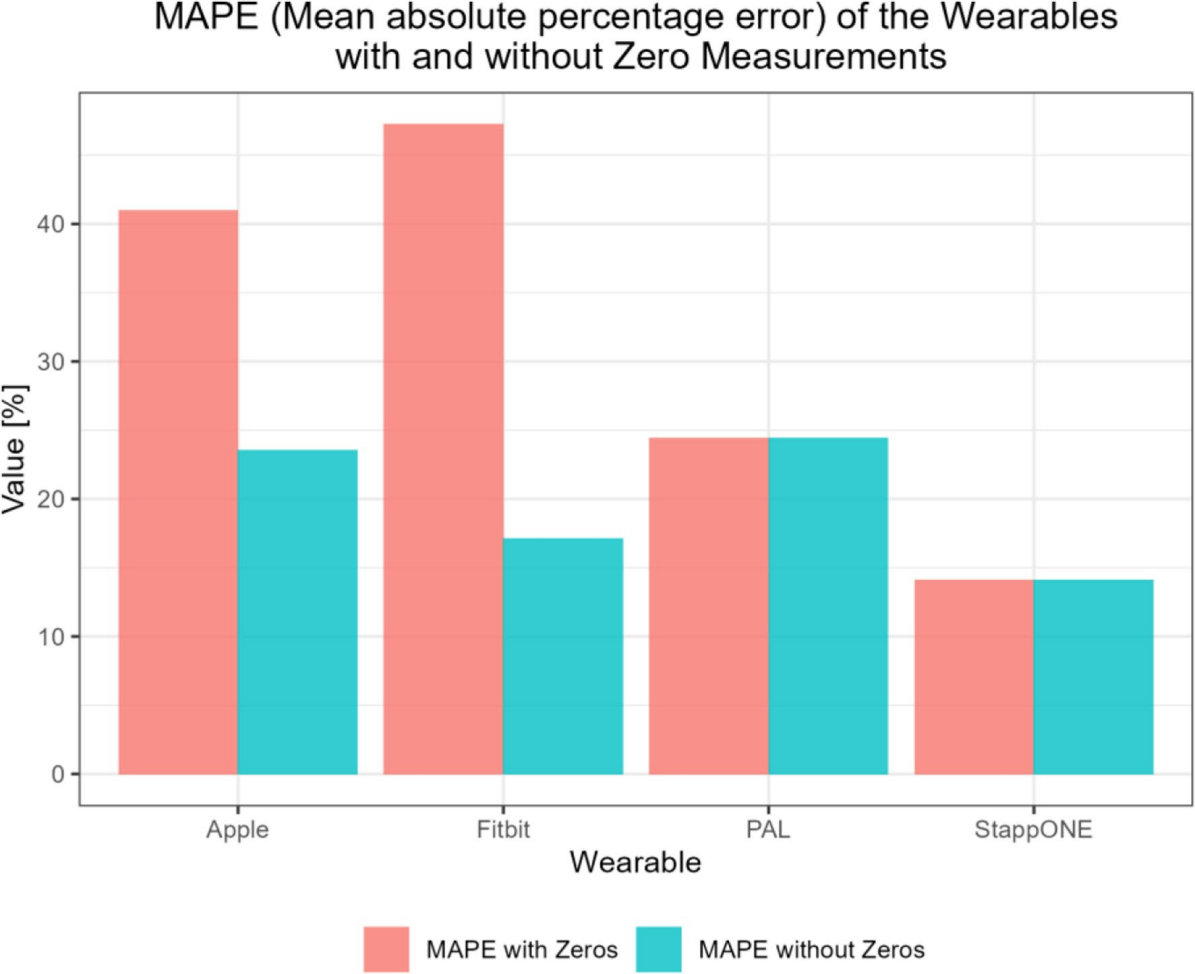
The bar plots show the mean absolute percentage error (MAPE) for the different Wearables. The Stappone v1.0 soles showed the lowest percentage error. After optimizing the results by eliminating the so-called zero values, the wrist-worn wearables showed a lower MAPE

Figure 2 shows the MAPE with and without error measurements. It shows that the StappOne Insole performs best both with and without zero measurements. When the zero measurements for the AppleWatch and Fitbit are ignored, there is a significant improvement in accuracy and a better result than with the activpal (Fig. 2).

### Predicted values of deviation

**Fig. 3:**
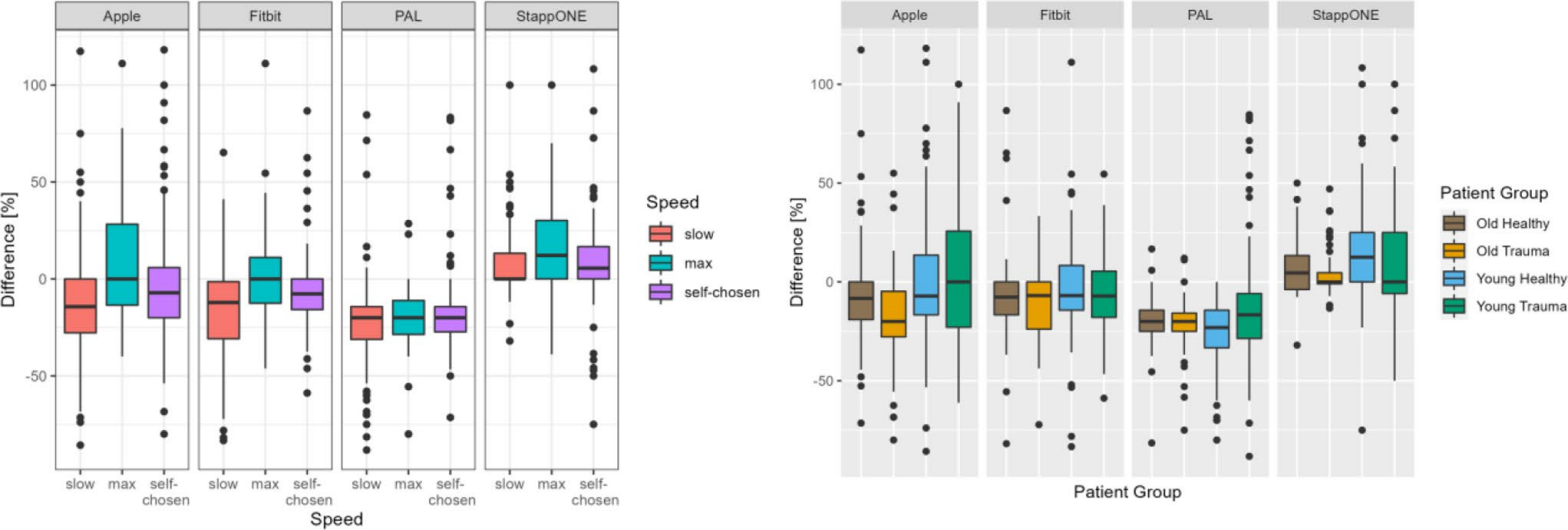
Optimizing the result by eliminating Zero Measurements. (**A**): Accuracy of the four different PAMs at different patient speeds, all results included. (**B**): The box plots describe the relative difference of steps recorded by the wearables to the actual number of steps depending on the patient group.Speed (Gl: slow gait speed; Gm: maximum gait speed, Gs: self-chosen gait speed).Patient Group (AG: Elderly (> 75 y.), healthy participants with free gait and no mobility impairment; AT Elderly (> 75 y.) patients with a lower limb injury or fracture; JG: Young (< 75 y.), healthy participants with free gait and no mobility impairment; JT Young (< 75 y.) participants with a fracture or injury to the lower limb that potentially alters the gait pattern)

The following plot show the values predicted by the model, assuming the average velocity (0.8 m/s - Fig. 3) for the influence variable not shown.

### Predicted probabilities of zero measurements

Finally, a general linear mixed model was fit for Apple and Fitbit, respectively, interpreting the zero measurements as a binomially distributed target variable.

**Fig. 4:**
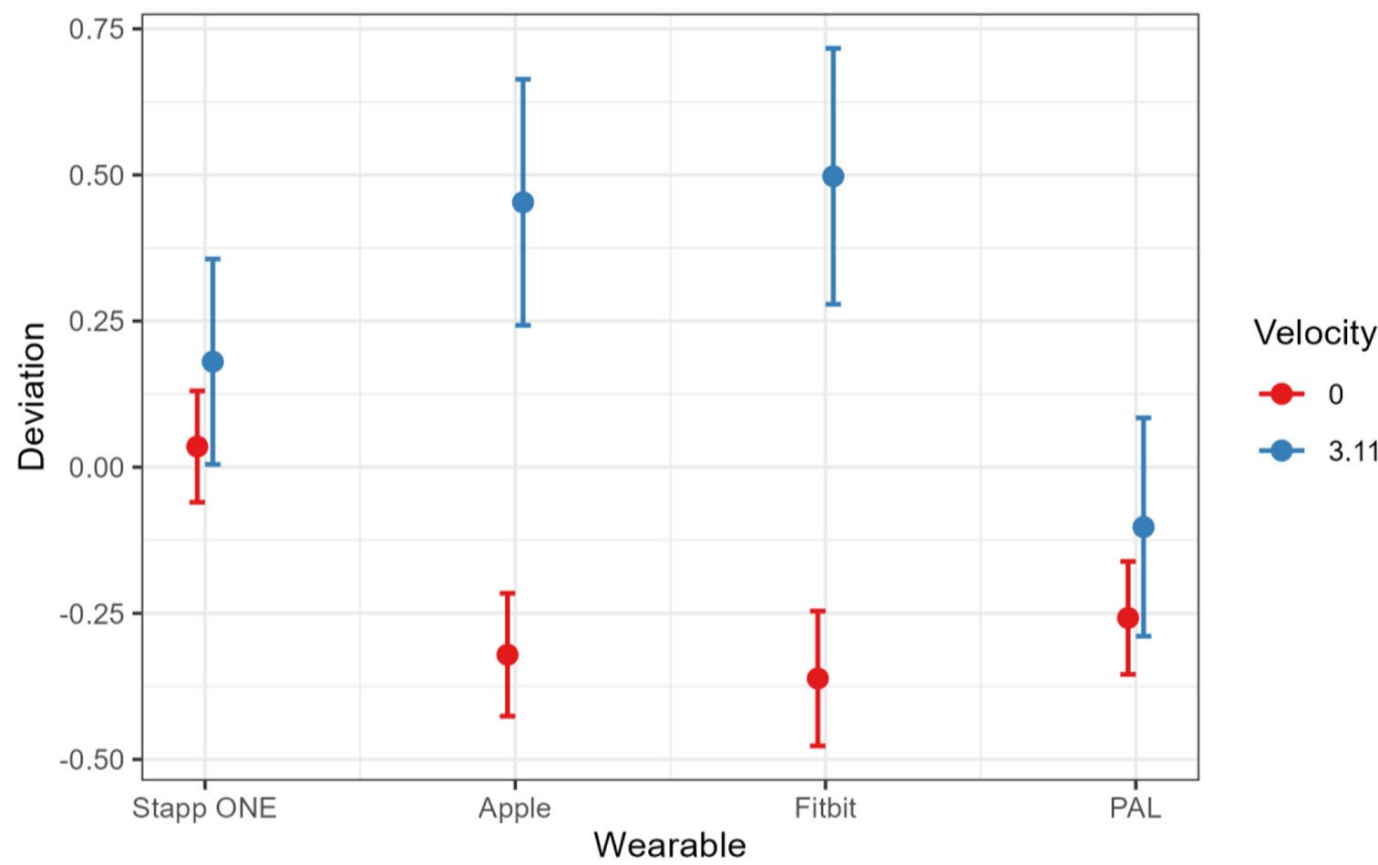
Predicted Values of deviation by velocity. The highest deviation is shown by the wrist worn devices AppleWatch and FitBit, the StappOne Insoles show the lowest deviation

**Fig. 5:**
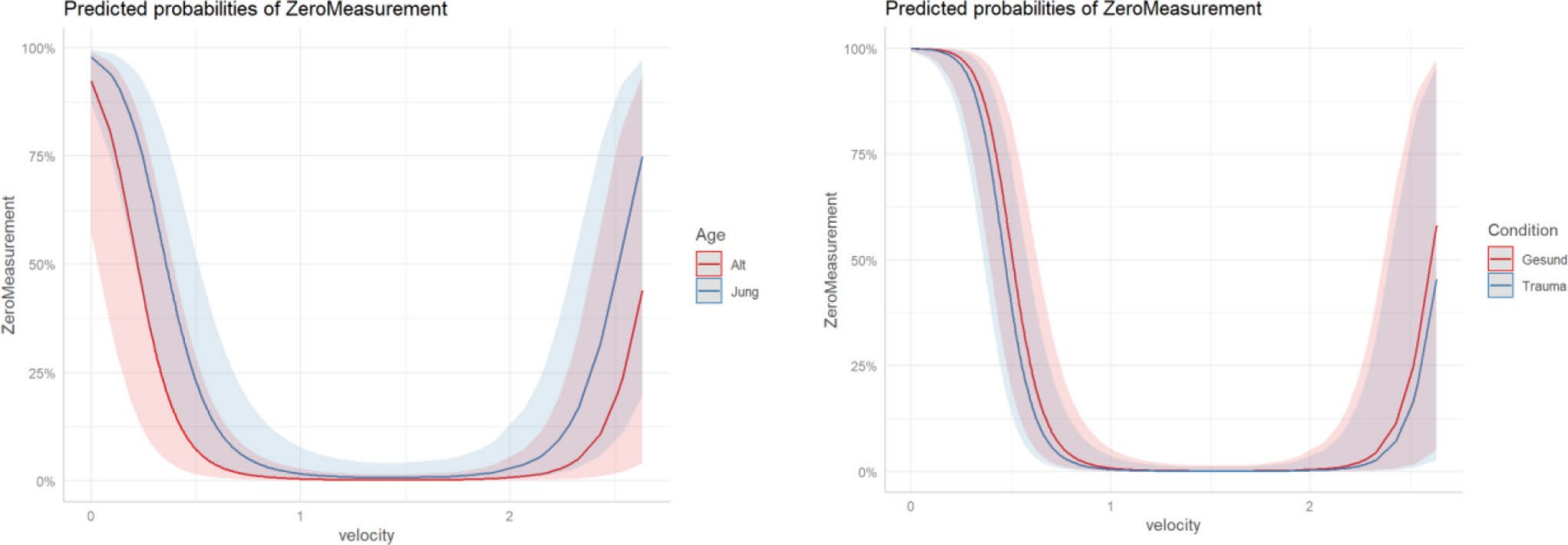
Predicted Probabilities of Zero-Measurements for Apple (left) and Fitbit (right) depending on the gear speed. At 1–2 m/s, the probability of a zero measurement is almost 0%..Generalized linear mixed model fit by maximum likelihood. Elderly (> 75 y.), Young (< 75 y.), healthy participants with free gait and no mobility impairment, patients with a lower limb injury or fracture

The results of such a model are shown below for Apple and Fitbit, respectively (Fig. 4 and 5), where the one-time gait speed of 4.8 m/s was interpreted as an outlier and discarded. It can be seen that for both devices, gait speed has the greatest influence on whether zero measurements occur, while age or trauma have a smaller influence.

## Discussion

The purpose of this study was to compare the step counting accuracy of different PAMs during a walking session at different walking speeds, with special attention to patients of different ages with walking impairment due to lower extremity injury or surgery. The results of this study indicate that all three accelerometer-based wearables underestimate step counts. Wrist-worn wearables have a significant number of zero measurements in slow-walking patients that are only slightly influenced by age or trauma. Insole measurements were found to have the highest validity, especially at slow walking speeds.

Most commercially available devices demonstrate high validity for step detection in young and healthy subjects [21–23]. Across devices, validity varies at different walking speeds, but most consumer activity trackers perform better at an average and vigorous walking speed than at a slower walking speed [24].Charge 3 Postoperative mobility tracking comes recently more into scientific focus [25]. It is feasible and patient-acceptable to continuously measure postoperative mobility, including step count and other gait characteristics, in orthogeriatric patients [17, 26]. However, the postoperative step count and gait speed are very low, so the validity of consumer activity trackers for such measurements must be critically questioned.

A major drawback is that most companies do not disclose algorithms that extract the accelerometer data, which complicates their scientific application. However, in a previous study, we demonstrated that optimized algorithms allow for much more accurate results in slow gate speeds of elderly people [13]. One promising avenue for addressing the challenges observed with step detection algorithms is the pioneering work conducted by collaborative initiatives such as the Mobilise-D consortium (https://mobilise-d.eu) [27]. These efforts have advanced the development of standardized algorithms and digital biomarkers to improve mobility assessments in clinical and real-world settings. By fostering multidisciplinary cooperation, Mobilise-D provides a framework for validating wearable devices and optimizing their accuracy across diverse populations, including those with impaired gait patterns [28, 29]. Such initiatives highlight the importance of integrating cutting-edge algorithmic research with clinical needs, paving the way for the next generation of mobility monitoring technologies.

In summary, the tested commercial accelerometer-based wearables need to further improve the algorithms for slower gait speeds to be usefully applied in orthopedic or patient treatment. The wrist-worn wearables tested are not useful in a slow-walking patient population due to multiple zero measurements likely generated by minimal arm movement. However, even without zero measurements, the mean percentage error of all accelerometer-based PAMs is about 20%. In contrast, insole measurements have a higher validity and are a possible alternative, e.g. during hospitalization or when accelerometer algorithms optimized for slow gait speeds are not available. In an initial approach, pressure-based sensor soles were also suitable for detecting frailty in orthogeriatric patients. Thanks to the large amount of data collected and the use of machine learning, a better prediction can be made than with the previously used scores [17, 30].

This study has several limitations. It should be noted that the walking distance was relatively short and did not include any special tasks or free-living parkour. However, the distances covered during an inpatient stay or after an injury are very limited [31]. It is therefore all the more important that the algorithm’s reaction times are short and yet precise. In addition, many studies are not comparable to our results because they are based on different devices or different software versions. This must always be taken into account, as regular changes in the software can have a considerable influence on the measurements.

## Conclusion

Accelerometer-based wearables require significant algorithmic improvements to address the challenges of slow and pathological gait patterns, especially for clinical populations. The frequent occurrence of zero measurements with wrist-worn devices highlights their limited utility in patients with restricted arm swing or lower extremity impairments. This limitation is particularly relevant in clinical settings, where patients often rely on mobility aids such as walkers or rollators, which inherently reduce arm movements. These factors must also be considered when using wrist-worn devices for rehabilitation, as they may lead to inaccurate assessments. To ensure effective rehabilitation outcomes, it is crucial to match the device capabilities to the specific needs and characteristics of the patient. Pressure-based systems, while more accurate, face practical challenges for everyday use, underscoring the need for tailored solutions that balance accuracy with usability.

## Data Availability

Available upon request by the corresponding author.
